# Bridge Condition Assessment Using *D* Numbers

**DOI:** 10.1155/2014/358057

**Published:** 2014-02-13

**Authors:** Xinyang Deng, Yong Hu, Yong Deng

**Affiliations:** ^1^School of Computer and Information Science, Southwest University, Chongqing 400715, China; ^2^Institute of Business Intelligence and Knowledge Discovery, Guangdong University of Foreign Studies, Sun Yat-sen University, Guangzhou 510006, China; ^3^School of Engineering, Vanderbilt University, Nashville, TN 37235, USA

## Abstract

Bridge condition assessment is a complex problem influenced by many factors. The uncertain environment increases more its complexity. Due to the uncertainty in the process of assessment, one of the key problems is the representation of assessment results. Though there exists many methods that can deal with uncertain information, however, they have more or less deficiencies. In this paper, a new representation of uncertain information, called *D* numbers, is presented. It extends the Dempster-Shafer theory. By using *D* numbers, a new method is developed for the bridge condition assessment. Compared to these existing methods, the proposed method is simpler and more effective. An illustrative case is given to show the effectiveness of the new method.

## 1. Introduction

The condition of bridge must be monitored and assessed periodically for the aim of keeping safety and facilitating maintenance. However, the bridge condition assessment is very complex because a bridge is composed of many components, and the relative importance of different components is different. The problem of bridge condition assessment has aroused the concern of more and more researchers [1–9].

At present, there exists many methods for the bridge condition assessment, such as evidential reasoning approach [10], interval reliability based method [11], and systematically validated finite-element model [12]; see also [13]. Generally, due to the complexity of bridge condition assessment, hierarchical method [14] is used first to establish a hierarchical model for the assessment and then aggregate the assessment results on different factors to obtain an overall assessment for a bridge. During this assessment process, due to lack of precise instrument and the limitation of cost, the assessment result of each factor is often given by bridge monitor through the visual observation according to his previous experience. Therefore, it is inevitable to involve the subjective judgement of human beings. The assessment results are full of uncertainty.

So the representation of assessment results under uncertain environment is a basic and key problem for the bridge condition assessment. Usually, the assessment results are represented by numerical ratings [5–7]. The bridge monitor assigns a rating to the assessment object. However, due to the complexity of environment, in many cases it may be difficult for the bridge monitor to assign a certain rating to an assessment object with 100% confidence. In addition, the assessment result of an object may not be precisely represented by using only one assessment rating. In this situation, the representation of assessment results is a problem needing to be solved first of all. At present, there are some tools that can deal with the uncertain information, such as fuzzy numbers of fuzzy set theory [15] and belief function of Dempster-Shafer theory [16, 17]. These theories have been widely applied to many fields, such as job-shop scheduling [18], shortest path problem [19, 20], and supply chain management [21, 22]; see also [23–28]. But there exist some deficiencies in these theories. Specific to the bridge condition assessment, a simple and effective representation is necessary.

In [10], Wang et al. used a dualistic group of assessment grade, belief degree, to show the assessment results. In fact, to our opinion the representation of assessment grade, belief degree, is a kind of *D* numbers, which will be introduced in this paper. *D* numbers [29–31] are a new representation of uncertain information. It is an extension of basic probability assignment of Dempster-Shafer theory. Though the form of *D* numbers also uses dualistic group; however, there exists essential difference between *D* numbers and the structure assessment grade, belief degree. In *D* numbers, these numerical ratings, such as 5, 6, and 7, are not absolute mutual exclusive. It is easy to understand that the nonexclusive hypotheses are very useful for the bridge condition assessment. In this paper, based on the *D* numbers, a new method for the bridge condition assessment is developed. The proposed method is simple but effective and it can be easily used in the bridge condition assessment and other fields. An illustrative case is given to show its effectiveness.

The remainder of this paper is organized as follows. A brief introduction about Dempster-Shafer theory is represented in Section 2. Then, the concept of *D* numbers and new bridge condition assessment method is depicted in Section 3. After that, an illustrative example is presented in Section 4. Finally, conclusions are given in Section 5.

## 2. Preliminaries

Dempster-Shafer theory of evidence [16, 17], also called Dempster-Shafer theory or evidence theory, is used to handle uncertain information. It is first proposed by Dempster and then developed by Shafer. This theory needs weaker conditions than Bayesian theory of probability, so it is often regarded as an extension of the Bayesian theory. As a theory of reasoning under the uncertain environment, Dempster-Shafer theory has an advantage of directly expressing the “uncertainty” by assigning the probability to the subsets of the set composed of *N* objects, rather than to each of the individual objects. The probability assigned to each subset is limited by a lower bound and an upper bound, which, respectively, measure the total belief and the total plausibility for the objects in the subset. What is more, Dempster-Shafer theory has the ability of combining pairs of bodies of evidence or belief functions to derive a combining evidence or belief function. With the ability of dealing with the uncertainty or imprecision embedded in evidence, Dempster-Shafer theory has been widely applied to many fields [32–44]. For completeness of the explanation, a few basic concepts are introduced as follows.

**Definition**

Let *Ω* be a set which is mutually exclusive and collectively exhaustive, indicted by

(1) $$\begin{array}{c} \Omega =\left\{E_{1},E_{2},\ldots ,E_{i},\ldots ,E_{N}\right\}. \end{array}$$

The set *Ω* is called frame of discernment. The power set of *Ω* is indicated by 2^*Ω*^, where

(2) 2Ω={∅,{E1},…,{EN},{E1,E2},…, {E1,E2,…,Ei},…,Ω}.

If *A* ∈ 2^*Ω*^, *A* is called a proposition.

**Definition**

For a frame of discernment *Ω*, a mass function is a mapping *m* from 2^*Ω*^ to [0,1], formally defined by

(3) $$\begin{array}{c} m:2^{\Omega }⟶\left[0,1\right] \end{array}$$

which satisfies the following condition:

(4) $$\begin{array}{c} m\left(\emptyset \right)=0,\;\;\underset{A\in 2^{\Omega }}{\sum }m\left(A\right)=1. \end{array}$$

In Dempster-Shafer theory, a mass function is also called a basic probability assignment (BPA) of the frame of discernment *U*. If *m*(*A*) > 0, *A* is called a focal element, the union of all focal elements is called the core of the mass function.

**Definition**

For a proposition *A*⊆*Ω*, the belief function Bel : 2^*Ω*^ → [0,1] is defined as

(5) $$\begin{array}{c} \text{Bel}\left(A\right)=\underset{B\subseteq A}{\sum }m\left(B\right). \end{array}$$

The plausibility function Pl : 2^*Ω*^ → [0,1] is defined as

(6) $$\begin{array}{c} \text{Pl}\left(A\right)=1-\text{Bel}\left(\bar{A}\right)=\underset{B\cap A\neq \emptyset }{\sum }m\left(B\right), \end{array}$$

where $\bar{A}=\Omega -A$.

Obviously, Pl(*A*) ≥ Bel(*A*); these functions Bel and Pl are the lower limit function and upper limit function of proposition *A*, respectively.

Consider two pieces of evidence indicated by two BPAs *m*_1_ and *m*_2_ on the frame of discernment *Ω*, Dempster's rule of combination is used to combine them. This rule assumes that these BPAs are independent.

**Definition**

Dempster's rule of combination, also called orthogonal sum, denoted by *m* = *m*_1_ ⊕ *m*_2_, is defined as follows:

(7) $$\begin{array}{c} m\left(A\right)=\{\begin{array}{cc} \frac{1}{1-K}\underset{B\cap C=A}{\sum }m_{1}\left(B\right)m_{2}\left(C\right), & A\neq \emptyset ;\\ 0, & A=\emptyset \end{array} \end{array}$$

with

(8) $$\begin{array}{c} K=\underset{B\cap C=\emptyset }{\sum }m_{1}\left(B\right)m_{2}\left(C\right), \end{array}$$

where *K* is a normalization constant, called conflict coefficient of two BPAs. Note that Dempster's rule of combination is only applicable to such two BPAs which satisfy the condition *K* < 1.

## 3. Proposed Method

In this section, a new method for the bridge condition assessment is introduced under the uncertain environment based on a new representation of uncertainty called *D* numbers [29–31].

### 3.1. *D* Numbers

In the frame of Dempster-Shafer theory, the basic probability assignment, defined on a mutually exclusive and collectively exhaustive set called frame of discernment, is used to express the uncertainty of judgement or environment. There exists some strong hypotheses on the frame of discernment and basic probability assignment.

First, the elements in the frame of discernment are required to be mutually exclusive. In many cases, however, the hypothesis is difficult to satisfy. In general, the frame of discernment is determined by experts. It is inevitable to involve human being's subjective judgements and uncertainty, for example, an assessment for a project; the representation of assessment is usually given by some numerical ratings, such as 1, 2, 3, 4, and 5. The exclusive hypothesis cannot be guaranteed precisely. Hence, the use of Dempster-Shafer theory is questioned to some degree. At present, there are already some studies about frame of discernment with nonexclusive hypotheses [45, 46].

Second, the sum of basic probability assignment must be equal to 1. It is a strict constraint so that the incomplete information is difficult to express by using basic probability assignment. Consider this situation that in a very complex environment, the evaluator does not have the overall judgement. The assessment is only on the basis of partial information. Therefore, it is possible that an incomplete BPA is obtained. From another perspective, in an open world the incompleteness may be reflected in the frame of discernment; the evaluator thinks that the frame of discernment does not contain all situations, so the emergence of an incomplete BPA is also reasonable. However, Dempster's rule of combination cannot handle the incomplete BPA. So the classical Dempster-Shafer theory is limited by the constraint.

In addition, there are some shortcomings in Dempster-Shafer theory, such as the computational complexity. In Dempster-Shafer theory, the uncertain information is represented by a basic probability assignment on the power set of problem domain and not by a probability on the the set of problem domain. Obviously, the computation effort of Dempster's rule of combination is not accepted when the number of framework is high to some degree. It is an often mentioned obstacle for the use of Dempster-Shafer theory [47–49].

So Dempster-Shafer theory has some inevitable deficiencies for the application in the real world. A valuable research is the extension of Dempster-Shafer theory. Intuitively, if some hypotheses of Dempster-Shafer theory have been removed reasonably, the ability of representing and handling uncertain information may be greatly improved. On the basis of this idea, a new representation of uncertain information is presented, which is called *D* numbers [29–31]. It is defined as follows.

**Definition**

Suppose the problem domain is indicated by a finite nonempty set which is denoted as *Ω*; *D* number is a mapping formulated by

(9) $$\begin{array}{c} D:2^{\Omega }⟶\left[0,1\right] \end{array}$$

with

(10) $$\begin{array}{c} \underset{B\subseteq \Omega }{\sum }D\left(B\right)\leq 1,\;\;\;D\left(\emptyset \right)=0, \end{array}$$

where *∅* is an empty set and *B* is a subset of *Ω*.

Note that (i) compared to the frame of discernment in Dempster-Shafer theory, the elements of set *Ω* do not require to be mutually exclusive in *D* numbers and (ii) the sum of *D* numbers is not strictly equal to 1. If ∑_*B*⊆*Ω*_ *D*(*B*) = 1, the information is said to be complete; If ∑_*B*⊆*Ω*_ *D*(*B*) < 1, the information is said to be incomplete. An example is given to show the differences between mass function and *D* numbers.

Let a bridge be assessed with scale interval [0,100]. An expert gives a BPA to express his assessment:

(11) $$\begin{array}{c} m\left(\left\{q_{1}\right\}\right)=0.5\\ m\left(\left\{q_{2}\right\}\right)=0.4\\ m\left(\left\{q_{1},q_{2},q_{3}\right\}\right)=0.1, \end{array}$$

where *q*_1_ = [0,60], *q*_2_ = [61,80], and *q*_3_ = [81,100]. Note that the elements in set {*a*_1_, *a*_2_, *a*_3_} are not intersecting and therefore the set is a frame of discernment.

And another expert uses *D* numbers to express his assessment:

(12) $$\begin{array}{c} D\left(\left\{b_{1}\right\}\right)=0.5\\ D\left(\left\{b_{2}\right\}\right)=0.3\\ D\left(\left\{b_{1},b_{2},b_{3}\right\}\right)=0.1, \end{array}$$

where *b*_1_ = [0,60], *b*_2_ = [45,75], and *b*_3_ = [65,100]. Obviously, the set of {*b*_1_, *b*_2_, *b*_3_} is not a frame of discernment due to the elements in the set of {*b*_1_, *b*_2_, *b*_3_} not being mutually exclusive. And because *D*({*b*_1_}) + *D*({*b*_3_}) + *D*({*b*_1_, *b*_2_, *b*_3_}) < 1, the information is incomplete. The example has shown the difference between mass function and *D* numbers.

Let *Ω* = {*b*_1_, *b*_2_,…, *b*_*i*_,…, *b*_*n*_} with *b*_*i*_ ∈ *R* and let *b*_*i*_ ≠ *b*_*j*_ if *i* ≠ *j*; a special *D* numbers can be expressed by

(13) D({b1})=v1D({b2})=v2⋮D({bi})=vi⋮D({bn})=vn

simple noted for *D* = {(*b*_1_, *v*_1_), (*b*_2_, *v*_2_),…, (*b*_*i*_, *v*_*i*_),…, (*b*_*n*_, *v*_*n*_)}, where *v*_*i*_ > 0 and ∑_*i*=1_^*n*^ *v*_*i*_ ≤ 1.

**Remark**

Permutation invariability: If there are two *D* numbers that *D*_1_ = {(*b*_1_, *v*_1_),…, (*b*_*i*_, *v*_*i*_),…, (*b*_*n*_, *v*_*n*_)} and *D*_2_ = {(*b*_*n*_, *v*_*n*_),…, (*b*_*i*_, *v*_*i*_),…, (*b*_1_, *v*_1_)}, then *D*_1_⇔*D*_2_.

**Remark**

Let *D* = {(*b*_1_, *v*_1_), (*b*_2_, *v*_2_),…, (*b*_*i*_, *v*_*i*_),…, (*b*_*n*_, *v*_*n*_)} be a *D* number; the integration representation of *D* is defined as

(14) $$\begin{array}{c} I\left(D\right)=\overset{n}{\underset{i=1}{\sum }}b_{i}v_{i}, \end{array}$$

where *b*_*i*_ ∈ *R*, *v*_*i*_ > 0 and ∑_*i*=1_^*n*^ *v*_*i*_ ≤ 1.

### 3.2. The Bridge Condition Assessment Based on *D* Numbers

In this paper, a new method for the bridge condition assessment based on *D* numbers is developed. Generally, it contains four phases.

#### 3.2.1. Build a Hierarchical Model for the Bridge Condition Assessment

In the real world, the condition of bridge is affected by many factors, such us fender system, bearing devices, and timber decay. It is impossible to find all potential factors and assess their influence to the bridge. It is very costly. So a general practice is to find the main factors and assess their influence to the bridge. Hence, it is necessary to collect these factors related to the bridge condition assessment as much as possible and then analyze these factors to identify the relative important factors, so that a hierarchical model can be established for the bridge condition assessment. In this field, some researchers have identified many factors related to the bridge condition assessment [5–8, 10, 50], these studies can provide some valuable references.

#### 3.2.2. Determine the Weight and Assessment Rating for Each Factor

Different factor plays different role in a bridge; some factors are more important than other factors. Hence, once a hierarchical model for the bridge condition assessment has been built, the weight of each factor in every layer should be determined according to the relative importance of factors. Some useful methods can be used in this work, such as analytic hierarchy process (AHP) method [14] and Delphi method [51].

In this phase, another task is to determine the assessment rating so that an assessment can be given to every factor. A lot of researchers have developed many standards. For example, a standard developed by Liang et al. [6] contains five ratings, namely, nondamage, light damage, moderate damage, severe damage, and unfit for service. Dunker and Rabbat [5] developed an assessment standard which contains nine grades, namely, failed condition, imminent failure condition, critical condition, serious condition, poor condition, fair condition, satisfactory condition, good condition, very good condition, and excellent condition. Here, a recommended assessment standard is New York BMS [5, 7] which contains seven ratings.  Table 1 shows these ratings and their meaning.

Then, the bridge monitor can assess the influence of every factor to the condition of bridge. It is worth noting that the assessment results are represented by using *D* numbers.

#### 3.2.3. Calculate the Integration Representation of the Assessment of Bottom Factors

In this phase, due to the assessment for bridge condition factors are represented by *D* numbers; it is necessary to use an operation on *D* numbers to process these assessment results. Here, based on the defined integration representation of *D* numbers, the assessment results of bottom factors are aggregated to real numbers. An example is given to show the process.

**Example**

**Figure 1:**
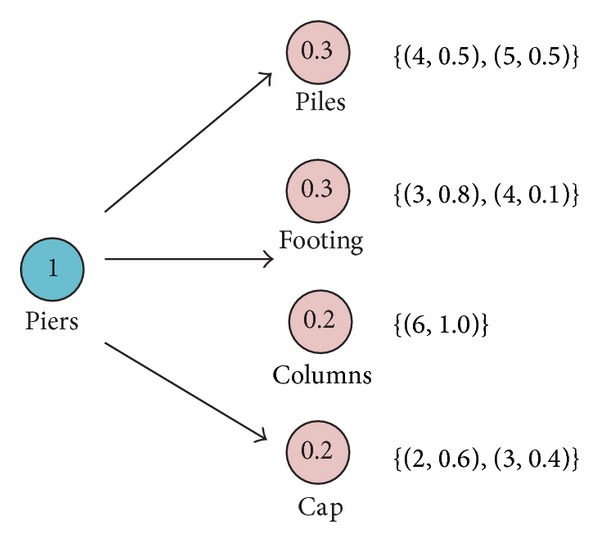
The integration of assessment results.

Piers are an important aspect for the condition of bridge. In general, it consists of many factors, such as piles, footing, columns, and cap. Assume the weights of factors are determined and the bridge monitor has assessed these factors based on the New York BMS condition ratings shown in Table 1.  Figure 1 shows the relative important relationship and assessment results.

According to (14), these assessment results represented by *D* numbers can be integrated as follows.

 For piles, *I*({(4,0.5), (5,0.5)}) = 4 × 0.5 + 5 × 0.5 = 4.5.
 For footing, *I*({(3,0.8), (4,0.1)}) = 3 × 0.8 + 4 × 0.1 = 2.8.
 For columns, *I*({(6,1.0)}) = 6 × 1.0 = 6.0.
 For cap, *I*({(2,0.6), (3,0.4)}) = 2 × 0.6 + 3 × 0.4 = 2.4.

#### 3.2.4. Aggregate the Assessment by Stepwise Weighing

At last phase, it is the aggregation of all assessment results by stepwise weighing to obtain an overall assessment for the bridge. Supposing a factor *F* contains *n* subfactors, indicated by *f*_*i*_, *i* = 1,2,…, *n*. The weight of each subfactor is *w*_*i*_ and the assessment result of *f*_*i*_ is *R*_*i*_. The overall assessment result of *F*, indicated by *R*_*F*_, is calculated by

(15) $$\begin{array}{c} R_{F}=\overset{n}{\underset{i=1}{\sum }}w_{i}R_{i}. \end{array}$$

Using this method, the overall bridge condition rating *R* can be derived so that the decision maker could make a right decision according to the condition of bridge.

## 4. A Case Study

In this section, an illustrative case recognized from [10] is given to show the process of applying the new method to the bridge condition assessment.

**Figure 2:**
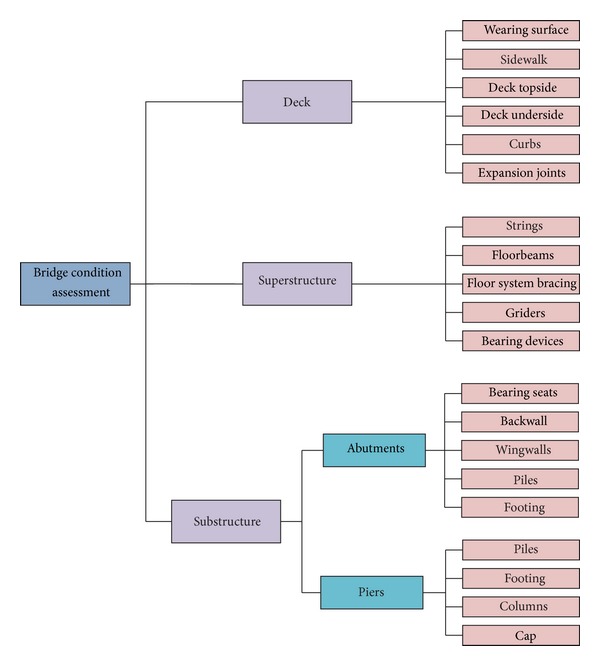
Hierarchical model for bridge condition assessment.

At the first phase, it needs to establish a hierarchical model for the bridge condition assessment. For simplicity, a hierarchical model developed by [10] is directly used in this phase.  Figure 2 shows the model for bridge condition assessment.

**Figure 3:**
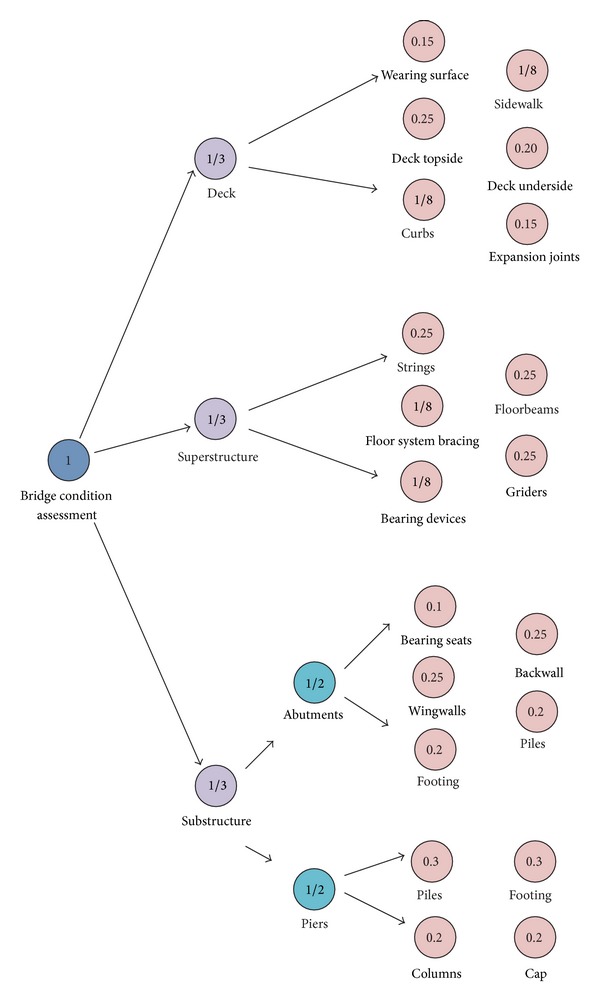
Weights of factors in each level.

At the second phase, the weight and assessment rating for each factor are determined. Here, the assignment of weight for every factor is shown in Figure 3. For the assessment ratings, a standard coming from [10] is adopted in this paper, as shown in Table 2.

According to the literature [10], the assessments given by experts for the condition of three bridges are given, as shown in Table 3.

At the third phase, the assessment results of bottom factors are integrated by using the integration interpretation of *D* numbers.  Table 4 shows the results of integrating the assessments for bottom factors.

At the last phase, the overall assessments of these three bridge can be obtained by stepwise weighing from subfactors to bridge's components, finally to the overall bridge. Tables 5–7 show the whole process.  Table 5 gives the results of weighing aggregation on subfactors.  Table 6 gives the results of weighing aggregation on components for each bridge.  Table 7 gives the overall assessments of condition for the three bridges.

According to the overall assessments of each bridge, for bridge 1, the overall assessment *R*_1_ = 4.568, which is between ratings 4 and 5 and closer to rating 5 (good condition). For bridge 2, the overall assessment *R*_2_ = 3.886, which is between ratings 3 and 4 but closer to rating 4 (fair condition). For bridge 3, the overall assessment *R*_3_ = 3.836, which is also between ratings 3 and 4 but closer to rating 4 (fair condition).

By assigning the overall assessment to their two adjacent ratings according to the numerical distance, we can obtain the similarity that the overall assessment belongs to each rating. For example, the overall assessment for bridge 1 is *R*_1_ = 4.568, due to distance  (*R*_1_, rating  5) = |4.568 − 5| = 0.432 and distance  (*R*_1_, rating  4) = 0.568, so similarity  (*R*_1_, rating  5) = 0.568 and similarity  (*R*_1_, rating  4) =  0.432. Consequently, the overall assessment for bridge 1 can be transformed into the forms of *D* numbers; thus, *D*_*b*_1__ = {(4,0.432), (5,0.568)}.

In a similar way, we can obtain

 similarity  (*R*_2_, rating  3) = 0.114 and similarity  (*R*_2_, rating  4) = 0.886,
 similarity  (*R*_3_, rating  3) = 0.164 and similarity  (*R*_3_, rating  4) = 0.836.

**Figure 4:**
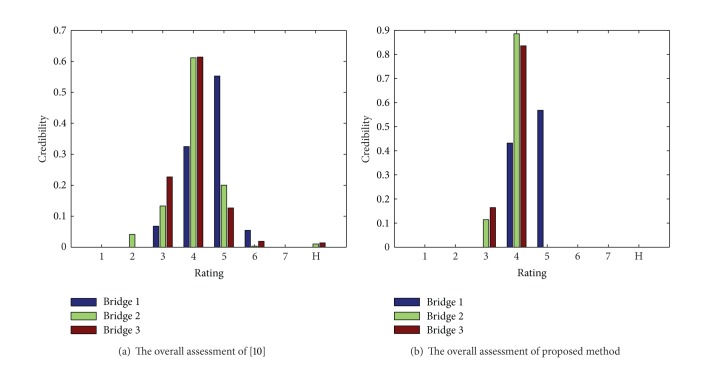
The comparison of different methods' assessment results.

Hence, *D*_*b*_2__ = {(3,0.114), (4,0.886)} and *D*_*b*_3__ = {(3,0.164), (4,0.836)}. For the sake of comparison, Figure 4 shows the comparison of assessment results obtained by the proposed method and that of Wang's method [10].

Obviously, the condition of these three bridges is bridge  1≻bridge  2≻bridge  3, where symbol ≻ represents “better than.” The proposed method has obtained identical assessment results with respect to the literature [10]. However, the proposed method is much simpler than the analytical evidence reasoning algorithm used in the literature [10]. Moreover, the *D* numbers are more effective to represent the uncertain subject assessments. Therefore, the proposed method shows its effectiveness for the bridge condition assessment.

## 5. Conclusions

In this paper, the bridge condition assessment is investigated under uncertain environment. At first, the representation of uncertain information is studied. By analyzing the shortcomings of Dempster-Shafer theory, a new representation of uncertain information, called *D* numbers, is presented. It is more effective to express the uncertainty. At second, based on the *D* numbers a new method for the bridge condition assessment is developed. The new method is simpler and more effective. An illustrative example has shown the new method's effectiveness.

## Figures and Tables

**Table 1:** The assessment ratings of New York BMS for bridge condition assessment.

Rating	Meaning
1	Potentially hazardous
3	Serious deterioration
5	Minor deterioration
7	Excellent or new condition
2, 4, 6	Between two adjacent ratings

**Table 2:** The hypothetical assessment ratings.

Rating	Meaning
1	Critical condition
2	Very poor condition
3	Poor condition
4	Fair condition
5	Good condition
6	Very good condition
7	Excellent condition

**Table 3:** The assessments given by experts for the condition of three bridges.

Bridge factors (relative weights)	Bridge 1	Bridge 2	Bridge 3
Deck (1/3)			
Wearing surface (0.15)	{(5, 1.0)}	{(2, 0.6), (3, 0.4)}	{(4, 0.7), (5, 0.3)}
Sidewalk (0.125)	{(5, 0.2), (6, 0.8)}	{(4, 0.3), (5, 0.6)}	{(3, 1.0)}
Deck topside (0.25)	{(4, 0.5), (5, 0.5)}	{(3, 0.7), (4, 0.3)}	{(4, 0.8), (5, 0.2)}
Deck underside (0.20)	{(4, 0.8), (5, 0.2)}	{(4, 0.2), (5, 0.8)}	{(3, 0.4), (4, 0.6)}
Curbs (0.125)	{(5, 1.0)}	{(3, 0.1), (4, 0.9)}	{(4, 1.0)}
Expansion joints (0.15)	{(5, 1.0)}	{(4, 1.0)}	{(3, 0.8), (4, 0.2)}
Superstructure (1/3)			
Stringers (0.25)	{(5, 0.8), (6, 0.2)}	{(4, 0.7), (5, 0.3)}	{(3, 0.9), (4, 0.1)}
Floorbeams (0.25)	{(3, 0.4), (4, 0.6)}	{(4, 0.6), (5, 0.4)}	{(4, 1.0)}
Floor system bracing (0.125)	{(4, 1.0)}	{(4, 0.8)}	{(3, 0.8), (4, 0.2)}
Girders (0.25)	{(4, 0.4), (5, 0.6)}	{(2, 0.3), (3, 0.7)}	{(4, 0.9)}
Bearing devices (0.125)	{(5, 0.6), (6, 0.4)}	{(4, 0.5), (5, 0.5)}	{(5, 0.3), (6, 0.7)}
Substructure (1/3)			
Abutments (1/2)			
Bearing seats (0.1)	{(4, 0.5), (5, 0.5)}	{(3, 0.4), (4, 0.6)}	
Backwall (0.25)	{(5, 1.0)}	{(4, 0.8), (5, 0.2)}	{(4, 1.0)}
Wingwalls (0.25)	{(4, 0.4), (5, 0.6)}	{(3, 0.3), (4, 0.6)}	{(4, 1.0)}
Piles (0.2)	{(3, 0.2), (4, 0.8)}	{(4, 0.4), (5, 0.6)}	{(3, 0.8), (4, 0.2)}
Footing (0.2)	{(4, 0.9), (5, 0.1)}	{(3, 0.1), (4, 0.9)}	{(3, 0.5), (4, 0.5)}
Piers (1/2)			
Piles (0.3)	{(4, 0.5), (5, 0.5)}	{(4, 1.0)}	{(5, 1.0)}
Footing (0.3)	{(3, 0.9), (4, 0.1)}	{(4, 1.0)}	{(4, 0.2), (5, 0.8)}
Columns (0.2)	{(5, 0.6), (6, 0.4)}	{(5, 1.0)}	{(4, 0.5), (5, 0.5)}
Cap (0.2)	{(4, 0.3), (5, 0.7)}	{(4, 0.8), (5, 0.2)}	{(3, 0.4), (4, 0.6)}

**Table 4:** Integration of the assessment results for bottom factors.

Bridge factors (relative weights)	Bridge 1	Bridge 2	Bridge 3
Deck (1/3)			
Wearing surface (0.15)	5.0	2.4	4.3
Sidewalk (0.125)	5.8	4.2	3.0
Deck topside (0.25)	4.5	3.3	4.2
Deck underside (0.20)	4.2	4.8	3.6
Curbs (0.125)	5.0	3.9	4.0
Expansion joints (0.15)	5.0	4.0	3.2
Superstructure (1/3)			
Stringers (0.25)	5.2	4.3	3.1
Floorbeams (0.25)	3.6	4.4	4.0
Floor system bracing (0.125)	4.0	3.2	3.2
Girders (0.25)	4.6	2.7	3.6
Bearing devices (0.125)	5.4	4.5	5.7
Substructure (1/3)			
Abutments (1/2)			
Bearing seats (0.1)	4.5	3.6	
Backwall (0.25)	5.0	4.2	4.0
Wingwalls (0.25)	4.6	3.3	4.0
Piles (0.2)	3.8	4.6	3.2
Footing (0.2)	4.1	3.9	3.5
Piers (1/2)			
Piles (0.3)	4.5	4.0	5.0
Footing (0.3)	3.1	4.0	4.8
Columns (0.2)	5.4	5.0	4.5
Cap (0.2)	4.7	4.2	3.6

**Table 5:** Weighing aggregation on subfactors.

Bridge factors (relative weights)	Bridge 1	Bridge 2	Bridge 3
Deck (1/3)			
Wearing surface	0.75	0.36	0.645
Sidewalk	0.725	0.525	0.375
Deck topside	1.125	0.825	1.05
Deck underside	0.84	0.96	0.72
Curbs	0.625	0.4875	0.5
Expansion joints	0.75	0.6	0.48
Superstructure (1/3)			
Stringers	1.3	1.075	0.775
Floorbeams	0.9	1.1	1.0
Floor system bracing	0.5	0.4	0.4
Girders	1.15	0.675	0.9
Bearing devices	0.675	0.5625	0.7125
Substructure (1/3)			
Abutments (1/2)			
Bearing seats	0.45	0.36	
Backwall	1.25	1.05	1.0
Wingwalls	1.15	0.825	1.0
Piles	0.76	0.92	0.64
Footing	0.82	0.78	0.7
Piers (1/2)			
Piles	1.35	1.2	1.5
Footing	0.93	1.2	1.44
Columns	1.08	1.0	0.9
Cap	0.94	0.84	0.72

**Table 6:** Weighing aggregation on components of bridges.

Bridge factors (relative weights)	Bridge 1	Bridge 2	Bridge 3
Deck (1/3)	4.815	3.7575	3.77
Superstructure (1/3)	4.525	3.8125	3.7875
Substructure (1/3)	4.365	4.0875	3.95

**Table 7:** The overall assessments of condition for the three bridges.

Bridge	Bridge 1	Bridge 2	Bridge 3
Assessment	4.568	3.886	3.836
Ranking	1	2	3
